# Balanced NPK fertilization enhances maize yield and shapes rhizosphere bacterial communities in purple soil: evidence from a ten-year field experiment

**DOI:** 10.1186/s12866-026-05142-0

**Published:** 2026-05-12

**Authors:** Song Guo, Mengying Liu, Zhigang Liu, Kun Chen, Mingjiang He, Yunliang Li, Xing Luo, Yuxian Shangguan, Xiangzhong Zeng, Yonghong Liu, Yiting Ouyang, Zijun Zhou, Yusheng Qin

**Affiliations:** 1 Institute of Agricultural Resources and Environment, Sichuan Academy of Agricultural Sciences, Chengdu, 610066 P.R. China; 2 Department of Soil Science, University of Saskatchewan, 51 Campus Drive, Saskatoon, SK S7N 5A8 Canada; 3 Global Institute for Food Security, University of Saskatchewan, Saskatoon, SK S7N 4L8 Canada; 4 Crop Research Institute, Sichuan Academy of Agricultural Sciences, Chengdu, 610066 P.R. China; 5 Environment-Friendly and Efficient Water-Saving Technology and Equipment for Hilly Agriculture Key Laboratory of Sichuan Province, Chengdu, 610066 P.R. China

**Keywords:** Maize productivity, NPK fertilization, Soil nutrient dynamics, Rhizosphere microbiome, Bacterial community structure

## Abstract

Balanced fertilization with nitrogen (N), phosphorus (P), and potassium (K) plays a pivotal role in sustaining maize productivity and shaping soil-microbe interactions. However, the integrated effects of long-term fertilization on crop performance, soil nutrient dynamics, and rhizosphere bacterial communities in purple soils remain unclear. In a 10-year field experiment in purple soil, conducted under a randomized complete block design (RCBD) with three blocks, we evaluated the effects of different fertilization regimes on maize growth and yield, soil physicochemical properties, and bacterial community composition using 16 S rRNA gene sequencing. Nitrogen-containing fertilizer treatments (NPK, NK, NP) markedly enhanced grain yield, shoot and root biomass, and root morphological traits compared with unfertilized (CK) and PK treatments, with balanced NPK fertilization delivering the highest performance. Fertilization significantly influenced soil nutrient availability, with NPK maintaining more stable nutrient levels over time. Microbial analyses revealed that rhizosphere communities were more responsive to fertilization than bulk soil communities, with N availability driving diversity shifts and community separation. Key taxa, including *Variovorax*, *Microscillaceae*, *Lysobacter*, and *Dyadobacter*, were enriched in N-fertilized soils and positively correlated with grain yield and N uptake. Collectively, these findings demonstrate that balanced NPK fertilization, particularly nitrogen input, enhances maize productivity by simultaneously improving soil fertility and fostering beneficial rhizosphere microbial assemblages, offering a basis of sustainable nutrient and microbiome management in purple soil.

**Supplementary Information:**

The online version contains supplementary material available at 10.1186/s12866-026-05142-0.

## Introduction

Maize (*Zea mays* L.) is a globally important cereal crop that provides carbohydrates, proteins, vitamins, and minerals essential for human nutrition [1]. Its adaptability to diverse climates and soils has made it one of the most widely cultivated crops worldwide, with the United States, China, and Brazil as the leading producers [2]. Beyond its nutritional value, maize serves as a cornerstone of the animal feed, bioethanol, and food processing industries, thereby supporting rural livelihoods and contributing substantially to national economies. Its high yield potential is pivotal for addressing global food security while simultaneously offering significant socio-economic benefits [3].

To maximize maize yields, various agronomic strategies have been implemented, including cultivar selection, efficient nutrient and water management, conservation practices such as no-tillage and crop rotation, and integrated pest, weed, and disease control [4]. Among these, fertilization management—particularly the application of nitrogen (N), phosphorus (P), and potassium (K)—is critical for optimizing yields [5].

Nitrogen, phosphorus, and potassium are indispensable macronutrients for maize growth. N and P are key components of ATP, nucleic acids, and chlorophyll, supporting essential processes such as energy transfer, genetic regulation, and photosynthesis. Specifically, N enhances chlorophyll synthesis, while P contributes to the regulation of metabolic stress [6]. Deficiencies in N and P cause stunted growth, leaf chlorosis, delayed maturity, and impaired root and seed development [7]. Meanwhile, K regulates osmotic balance, enzyme activation, and stress tolerance; its deficiency compromises stem structural integrity and reduces overall plant vigor [8]. Collectively, these macronutrients are vital for sustaining maize productivity and promoting agricultural sustainability.

Nitrogen fertilization alone accounts for up to 75% of maize yield improvements by enhancing N uptake and both nutrient- and water-use efficiency [9]. However, while excessive N inputs can negatively impact plant growth and environmental quality, whereas moderate reductions (10–20%) may sustain crop performance and improve rhizosphere microbiome health [10]. Phosphorus availability is often limited in soils due to strong binding with aluminum, iron, or calcium. In phosphorus-deficient soils, P fertilization has been reported to significantly increase maize yields and plant growth, including biomass and plant height, although the magnitude of these responses varies depending on soil properties and environmental conditions [11, 12]. Potassium application has been shown to improve maize yield and N uptake, but its effects on P uptake and yield remain inconsistent [13]. Overall, balanced NPK fertilization provides the greatest agronomic benefits, although the relative contribution of each nutrient varies across plant growth stages [14].

Beyond direct plant responses, fertilizer regimes profoundly influence the rhizosphere microbiome, which plays key roles in nutrient cycling, soil fertility, and plant health and productivity. Nitrogen fertilization alters microbial diversity, affecting functional groups such as nitrifying bacteria and arbuscular mycorrhizal fungi [15]. Slow-release N has been reported to increase both bacterial and fungal diversity, thereby improving maize yield [16]. Phosphorus fertilization similarly shapes rhizosphere communities, increasing the abundance of P-solubilizing bacteria that facilitate plant growth [17]. Although less studied, potassium fertilization and combined NPK inputs also reshape microbial communities in both rhizosphere and bulk soils, with downstream effects on nitrogen, phosphorus, and carbon cycling [18, 19].

Purple soil, classified as Regosols in the FAO soil system, is a distinctive soil type derived from Jurassic and Cretaceous purple rocks and widely distributed in Southwest China. These soils cover approximately 21.99 million hectares nationwide, with the majority located in the Sichuan Basin, where they span about 16 million hectares and extend into surrounding regions. Sichuan Province alone accounts for more than half of the total purple soil area [20]. Due to extensive erosion and long-term cultivation, purple soils are generally poorly developed and characterized by low fertility, particularly limited nitrogen and organic matter [21]. In addition, purple soils are predominantly located on sloping farmland in subtropical regions, where high temperatures and abundant rainfall accelerate soil erosion and nutrient loss. Combined with a high land reclamation index, these factors often result in relatively low levels of nitrogen, phosphorus, and organic matter, thereby constraining crop productivity [22].

Soil microbial communities play a key role in regulating nutrient cycling and maintaining soil fertility in these fragile agroecosystems. Previous studies have shown that purple soils harbor diverse microbial communities involved in organic matter decomposition and nutrient transformation, although their composition and activity are highly sensitive to nutrient management practices [23]. In particular, fertilization regimes, especially NPK inputs, can substantially reshape rhizosphere microbial communities and influence microbial-mediated nutrient availability in purple soils [24]. Consequently, fertilization remains a critical management practice for sustaining and improving crop yields in this region [25].

Despite considerable progress in understanding maize nutrient requirements, critical knowledge gaps remain regarding how different NPK fertilization regimes influence microbial communities in both rhizosphere and bulk soils of purple soil systems, and how these microbial shifts may ultimately affect maize performance.

To address these gaps, this study evaluated the effects of five fertilization regimes (CK, NPK, NK, NP, PK) on maize growth, root development, and soil microbial communities in purple soil. Specifically, this study aimed to: (1) Quantify how different fertilization regimes influence maize growth, root system architecture, and yield performance in purple soil. (2) Determine how N, P, and K fertilization shape the diversity, structure, and composition of microbial communities in bulk soil and the rhizosphere. (3) Identify microbial taxa that respond specifically to N, P, or K inputs and explore their potential associations with plant growth traits and soil nutrient availability. By linking fertilization management with shifts in soil microbial communities and plant performance, this study provides new insights into microbial community–associated nutrient management strategies for sustainable maize production in purple soils.

## Materials and methods

### Site description, sampling, and sample processing

This study was conducted as part of a long-term fertilization experiment, initiated in 2012, at Sichuan Agricultural Research Institute Modern Experimental Station in Deyang City, Sichuan Province, China. The experiment employed a maize monoculture (*Zea mays* L., cv. CD30) under five distinct fertilization regimes: NPK, NP, NK, PK and an unfertilized control (CK).

The NPK treatment received 300 kg N ha⁻¹, 90 kg P ha⁻¹, and 150 kg K ha⁻¹. The NP treatment received 300 kg N ha⁻¹ and 90 kg P ha⁻¹; the NK treatment received 300 kg N ha⁻¹ and 150 kg K ha⁻¹; the PK treatment included 90 kg P ha⁻¹ and 150 kg K ha⁻¹. Urea, superphosphate, and potassium chloride served as the N, P, and K sources, respectively. In treatments receiving P and/or K, phosphorus and potassium fertilizers were applied as basal fertilizers prior to sowing, broadcast and incorporated by rotary tillage. In treatments receiving N, Nitrogen fertilizer was applied in two equal splits: half as a basal application and the remaining half as a topdressing at the stem elongation stage, surface-broadcasted and incorporated into the soil. The CK treatment received no fertilizer.

The experiment was arranged as a randomized complete block design (RCBD) with three blocks, for a total of 15 experimental plots (5 treatments × 3 blocks). Standard high-yield agronomic practices recommended for the region were applied uniformly across all treatments. Climate data (air temperature, sunshine duration, and precipitation) during the maize growing season were obtained from the local meteorological station and are presented in Fig. S1.

Maize was sown annually in April and harvested in August (2012–2021). Each plot measured 4 m × 5 m (20 m²) and employed a planting configuration with 20 cm plant spacing and alternating wide-narrow row spacing (70 cm × 130 cm), corresponding to a planting density of 50,000 plants ha^− 1^. Grain yield was determined for each plot at a standardized moisture content of 14% and calculated according to the harvested plot area. Baseline soil samples (0–30 cm) were collected prior to the experiment to determine pH, organic matter, available nitrogen (AN), available phosphorus (AP), and exchangeable potassium (AK) (Table S1). Following the methods described by Van et al. (1990), soil pH was determined potentiometrically in a 1:2.5 (w/v) soil–water suspension [26]. Organic matter was measured using the Walkley–Black dichromate oxidation method [27]. Available nitrogen was extracted with 2 M KCl and quantified by the Kjeldahl method to determine inorganic N (NH₄⁺ and NO₃⁻). Available phosphorus was extracted by the Olsen method (0.5 M NaHCO₃, pH 8.5) for neutral and calcareous soils, and P concentration was determined colorimetrically using the molybdenum blue method. Exchangeable potassium was extracted using 1 M ammonium acetate (pH 7.0) and measured by flame photometry. These soil properties were subsequently monitored after harvest in 2014, 2016, 2018, 2020, and 2021 for each plot.

In 2021, soil samples were collected at the V10 stage to characterize rhizosphere and bulk soil microbial communities after long-term exposure to different nutrient management treatments. Soil blocks (at dimensions of 60 cm × 40 cm × 30 cm, length × width × depth) were collected, and soil adhering directly to the roots (1–2 mm) was defined as rhizosphere soil, obtained by gently brushing off the roots. Bulk soil and rhizosphere soil samples were collected from each plot in duplicates. The duplicates were combined, resulting in a total of 15 bulk soil and 15 rhizosphere soil samples. All samples were homogenized, subsampled into 2 ml microtubes, and stored at -80 °C for subsequent DNA extractions and microbiota analysis.

At both the V10 stage and physiological maturity in 2021, three maize plants per plot were sampled. Plants were separated into roots, stems, and leaves to determine dry weight and concentrations. At the V10 stage, plant traits measured included root dry weight (RDW), root total length (RTL), root surface area (RSA), root average diameter (RAD), root total volume (RTV), and shoot concentration of nitrogen (SN), phosphorus (SP), and potassium (SK). Roots were rinsed under running water to remove debris. Segments of root were placed in water within an acrylic tray and scanned using an EPSON Perfection V850 scanner. The resulting images were processed with WinRHIZO software (Regents Instruments Inc.) to determine RTL, RSA, RAD, and RTV [28]. Following scanning, roots were heated at 105 °C for 30 min, then dried in an oven at 65 °C for 72 h to obtain RDW. At harvest, grain nitrogen (GN), phosphorus (GP), and potassium (GK) concentrations were analyzed, along with grain yield and relative yield, calculated as: (mean yield of a given treatment / mean yield of the NPK treatment) × 100%. Plant nitrogen concentration was determined by the Kjeldahl method after sulfuric acid‑hydrogen peroxide digestion, phosphorus was measured colorimetrically using the molybdenum blue method following the same digestion procedure, and potassium concentration was quantified by flame photometry from the same digest solution [28]. Additionally, soil properties, including pH, organic matter content, and available nitrogen (AN), phosphorus (AP), and potassium (AK), were analyzed for both bulk and rhizosphere soils collected at the V10 stage.

### DNA extraction and amplicon sequencing

DNA was extracted from 0.25 g of bulk soil and rhizosphere soil using the TGuide S96 Magnetic Soil /Stool DNA Kit (Tiangen Biotech Co., Ltd.) following the manufacturer’s protocol. DNA concentration was quantified using the Qubit dsDNA HS Assay Kit and Qubit 4.0 Fluorometer (Invitrogen, Thermo Fisher Scientific, Oregon, USA). Primer set 338 F (5’- ACTCCTACGGGAGGCAGCA-3’) and 806R (5’- GGACTACHVGGGTWTCTAAT-3’) were used to amplify the V3-V4 region of the 16 S rRNA gene from the genomic DNA extracted per sample. Both the forward and reverse 16 S primers were tailed with sample-specific Illumina index sequences to allow for deep sequencing. The PCR was performed in a total reaction volume of 10 µL: DNA template 5–50 ng, 338 F (10 µM) 0.3 µL, 806R (10 µM) 0.3 µL, KOD FX Neo Buffer 5 µL, dNTP (2 mM each) 2 µL, KOD FX Neo 0.2 µL, ddH_2_O up to 10 µL. The PCR conditions were as follows: an initial denaturation at 95 °C for 5 min, followed by 25 cycles of denaturation at 95 °C for 30 s, annealing at 50 °C for 30 s, extension at 72 °C for 40 s, and a final step at 72 °C for 7 min. The total of PCR amplicons was then purified with Agencourt AMPure XP Beads (Beckman Coulter, Indianapolis, IN) and quantified using the Qubit dsDNA HS Assay Kit and Qubit 4.0 Fluorometer (Invitrogen, Thermo Fisher Scientific, Oregon, USA). After individual quantification and standardized to 4 nM, 5 µL of amplicons per sample were pooled into a 1.5 mL microcentrifuge tube for sequencing with Illumina NovaSeq 6000 platform (Illumina, Santiago CA, USA).

### Bioinformatics and statistical analysis

The bioinformatics analysis of this study was performed in QIIME2 v. 2022.8 [29]. Specifically, identification and removal of primer sequences and adapters were processed by Cut adapt version 3.1 [30]. The forward and reverse reads were then joined by the Vsearch plug-in function “merge-pairs” [31]. Following this, Deblur [32] was used on the joined sequence reads, where sequences were truncated at a length of 444, to keep reads with a minimum quality score of 30. The high-quality reads in Amplicon Sequence Variants (ASVs) [33] format generated from the above steps were used in the following analysis. The SILVA database (Silva_138) was trained as the classifier [34] to classify the taxonomy of the above-generated ASVs. The processed sequence data, including the bacterial feature table and taxonomy classification table, were subsequently imported into R 4.2.3 for statistical analyses and visualization.

Plant performance and soil traits were analyzed. Differences among treatments were assessed by one-way analysis of variance (ANOVA), followed by Tukey’s post hoc test for multiple comparisons. For bacterial community analysis, the R packages vegan [35] and phyloseq [36] were used. Alpha diversity analysis in bulk soil and rhizosphere soil was performed using the Shannon diversity index on normalized samples based on the minimum total count observed across all samples. Beta-diversity based Principal Component Analysis (PCA) was conducted on centered log-ratio (CLR)-transformed data, focusing on taxa present in at least 80% of all samples. Permutational multivariate analysis of variance (PERMANOVA) was performed thereafter, to detect fertilizer treatment effects and sample compartment (bulk soil vs. rhizosphere soil) on bacterial beta diversity. Relative abundance in bulk soil and rhizosphere soil was conducted to profile the composition of bacterial taxa at phylum level. The nine most abundant phyla were presented individually, while the remaining low-abundance phyla were combined into a single category designated as “Others”. To identify bacterial taxa with different abundance under different fertilizer additions, Analysis of Compositions of Microbiomes with Bias Correction 2 (ANCOM-BC2) was performed [37]. Finally, Spearman correlation analysis was performed to link rhizosphere ASVs with plant traits and soil properties measured in 2021.

## Results

### Impact of different fertilizer treatments on maize growth and field performance

**Fig. 1:**
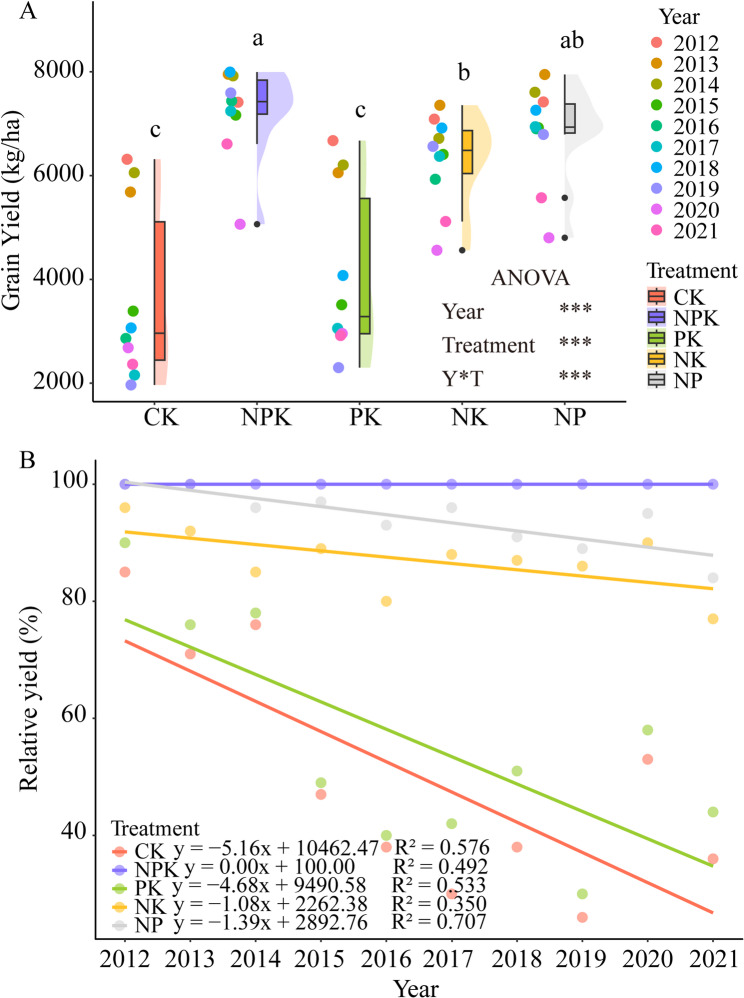
Grain yield (**A**) and relative yield (**B**) of maize under different fertilizer treatments from 2012 to 2021. Different lowercase letters indicate significant differences among treatments at *p* < 0.05 based on Tukey’s multiple comparison following ANOVA. ***indicates a highly significant effect (*p* < 0.001) based on ANOVA (*n* = 10)

Maize grain yield was significantly affected by year, fertilizer treatment, and their interaction over the 10-year period (2012–2021; Fig. 1A). The NPK treatment achieved the highest average yield (7,239 kg ha⁻¹), followed by NP (6,816 kg ha⁻¹), which did not differ significantly from NPK. The NK treatment averaged 6,302 kg ha⁻¹, a value significantly lower than that of the NPK treatment. The PK (4,070 kg ha⁻¹) and CK (3,654 kg ha⁻¹) treatments resulted in the lowest yields, while they were not significantly different from each other, both were significantly lower than all other fertilized treatments.

Notably, yield differences were not statistically significant in the first year of the study (2012). However, from 2013 onward, the PK and CK treatments produced significantly lower yields than NPK. Starting in 2019, the yields of NP and NK also became significantly lower than those of NPK, although they remained substantially higher than the PK and CK groups (Fig. S2). Long-term nutrient deficiency resulted in a progressive decline in productivity. Relative to the NPK, the average annual yield decreases were 1.08% for NP, 1.39% for NK, 4.68% for PK, and 5.16% for CK. Accelerated yield declines were observed after four years (post-2015), with the severity of the yield reduction ranked as N deficiency > P deficiency > K deficiency (Fig. 1B).

**Fig. 2:**
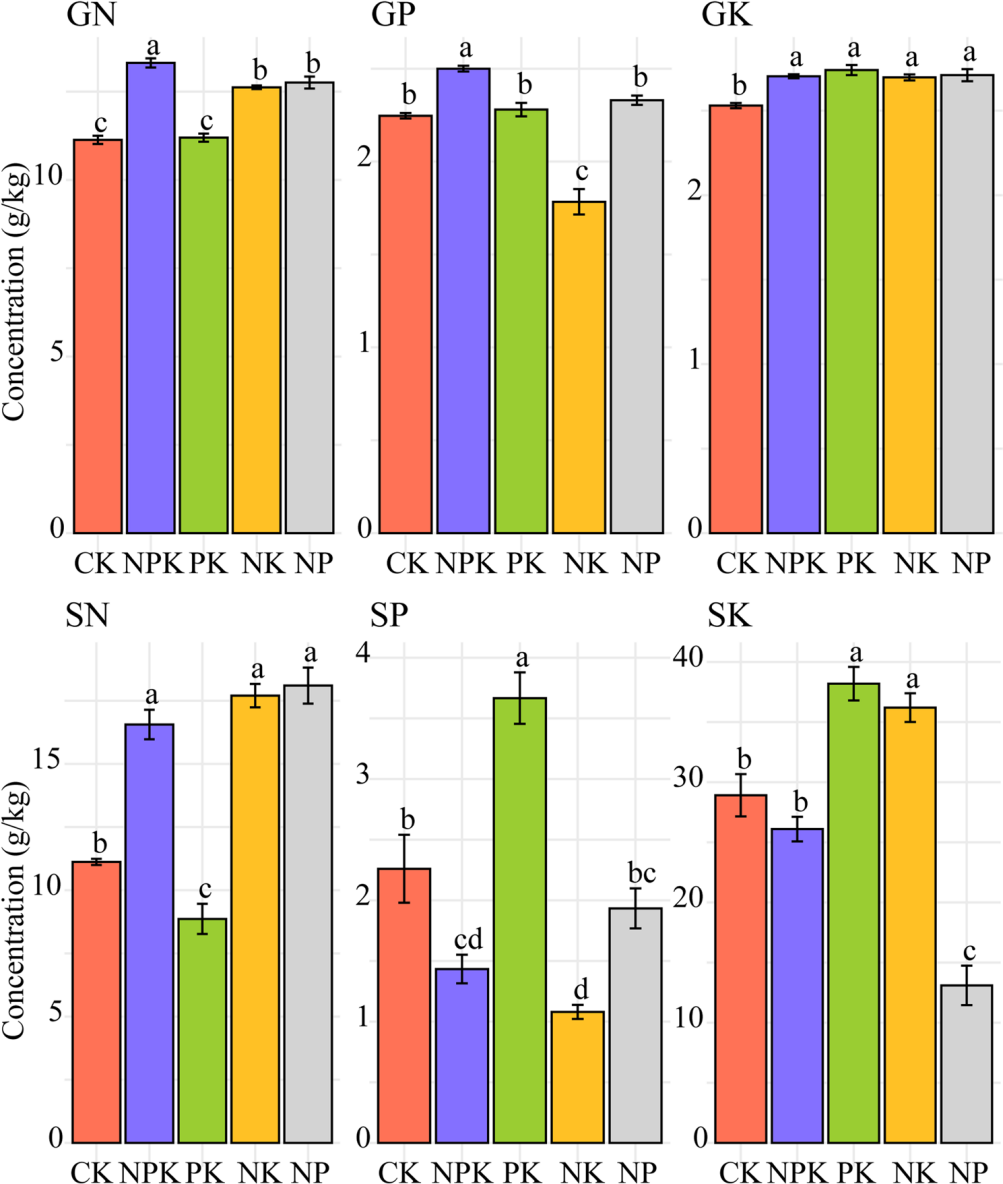
Grain (GN, GP, GK) and shoot (SN, SP, SK) concentrations of N, P, and K in maize under different fertilizer treatments. Values are presented as mean ± SD (*n* = 3). Different lowercase letters indicate significant differences among treatments at *p* < 0.05 based on Tukey’s post hoc test multiple comparison test

Different fertilization treatments significantly affected maize growth and nutrient accumulation. Balanced NPK fertilization resulted in the highest grain nitrogen (GN) and phosphorus (GP) contents (Fig. 2). Compared with CK, GN increased by 19.6% under NPK, 14.6% under NP, and 13.4% under NK, whereas no significant differences were observed between the PK and CK treatments. Similarly, GP was 11.1% higher under NPK than CK, while the NK significantly reduced GP by 20.9% relative to the control. Grain potassium (GK) content was significantly higher than in CK in all fertilized treatments, with increases ranging from 6.7% to 8.3%, although no significant differences were detected among the fertilized treatments (Fig. 2). Nitrogen-containing treatments (NPK, NP, and NK) significantly increased shoot nitrogen (SN) concentrations relative to CK by 48.9–62.8%, with no significant differences observed among these three treatments. In contrast, shoot phosphorus (SP) was highest under PK fertilization, showing a 62.4% increase compared with CK, whereas NK resulted in the lowest SP, which was 52.2% lower than CK. Shoot potassium (SK) concentrations were highest in the PK and NK treatments, increasing by 31.0% and 24.1% relative to CK, respectively. Conversely, the potassium-deficient NP treatment showed a significant reduction in SK, reaching only 44.8% of the levels observed in the CK (Fig. 2).

**Fig. 3:**
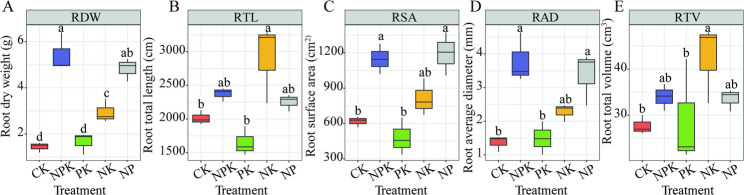
Root performance under different fertilizer treatments. **A** Root dry weight (RDW), **B** Root total length (RTL), **C** Root surface area (RSA), **D** Root average diameter (RAD) and (**E**) Root total volume (RTV). Different lowercase letters indicate significant differences among treatments at *p* < 0.05 based on Tukey’s post hoc test multiple comparison test (*n* = 3)

Nitrogen-containing treatments (NPK, NK, and NP) markedly enhanced root dry weight (RDW) compared with the CK, with increases of 27.8-, 10.5-, and 2.35-fold, respectively, whereas PK did not differ significantly from CK. Among all treatments, NPK produced the most robust root growth performance (Fig. 3A). Root growth under NP ranked second, followed by NK, while PK and CK showed the lowest values. Root morphology also varied significantly across fertilization regimes. Root total length and root total volume were greater under N-containing treatments than under PK or CK, increasing by 12–45% and 21–54%, respectively (Fig. 3B and E). Root surface area was highest in NP and NPK treatments, reaching 1,196 and 1,148 cm², respectively, representing increases of 93.5% and 85.8% compared with CK (Fig. 3C). Root diameter was greatest under NPK, increasing by 179% and 153% relative to CK and PK, respectively (Fig. 3D).

### Soil nutrient dynamics and bacterial diversity in bulk and rhizosphere soils

**Fig. 4:**
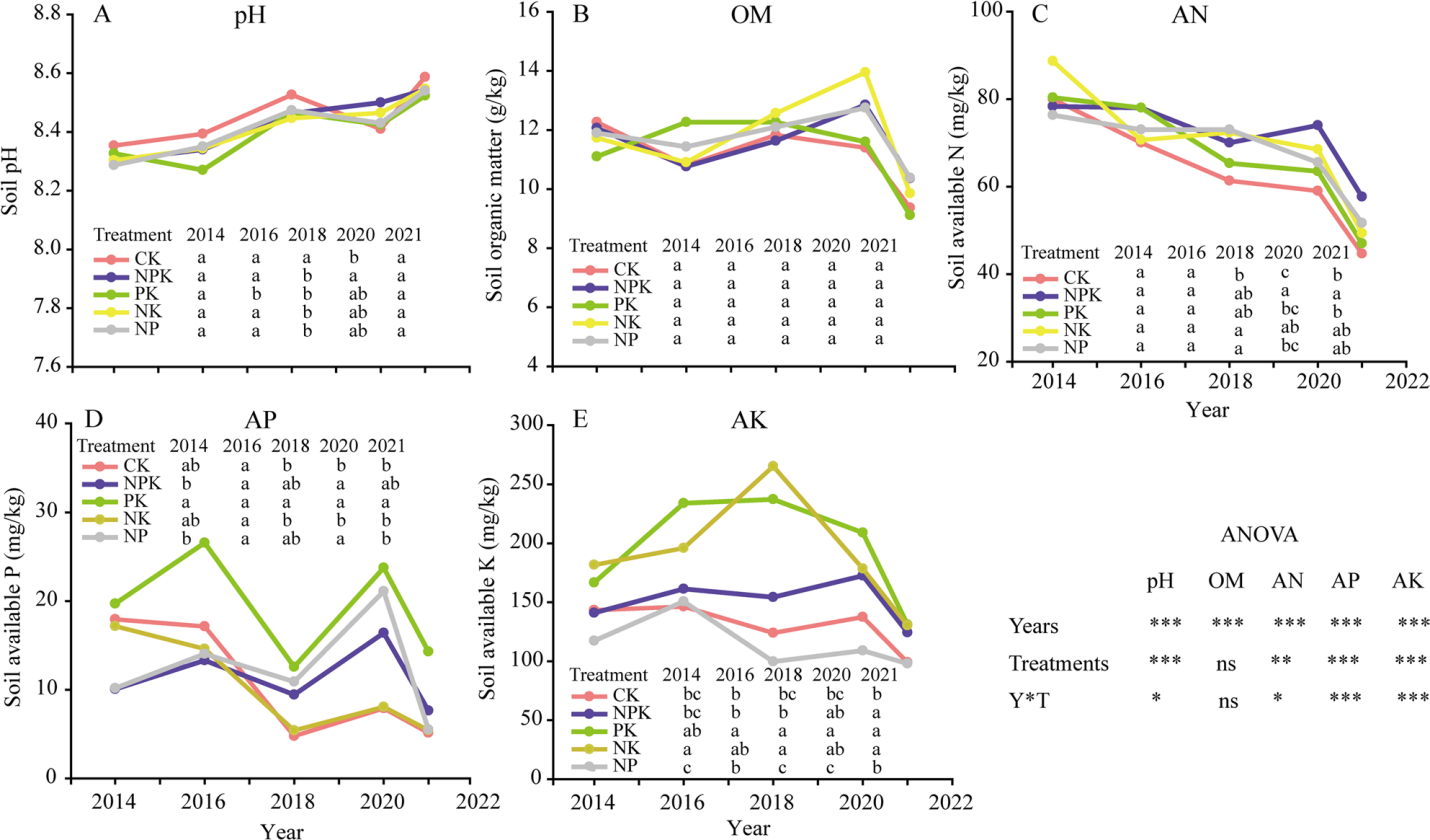
Soil nutrient status under different fertilizer treatments: no fertilizer (CK), nitrogen and phosphorus (NP), nitrogen and potassium (NK), phosphorus and potassium (PK), and nitrogen, phosphorus, and potassium (NPK). **A** pH, **B** Organic matter (OM), **C** Available N (AN), **D** Available P (AP), and (**E**) available K (AK). Different lowercase letters indicate significant differences among treatments at *p* < 0.05 based on Tukey’s post hoc multiple comparison test following ANOVA. ns: not significant, *p < 0.05, **p < 0.01, and ***p < 0.001 indicate highly significant effect based on ANOVA (*n* = 3)

Long-term monitoring of soil nutrient dynamics from 2012 to 2021 revealed that year, fertilizer treatment, and their interaction significantly affected soil pH, available nitrogen (AN), available phosphorus (AP), and available potassium (AK) (Fig. 4). Soil pH remained weakly alkaline and increased gradually over the 10-year period (Fig. 4A). Soil organic matter declined overall, decreasing by 10–29% in 2021 compared with 2014 (Fig. 4B), with slightly higher values under NPK and PK than CK in most years, although the differences were not significant. AN decreased by 30–50% during the experimental period, with the NPK treatment consistently maintaining higher AN levels than other treatments; after 2018, depletion accelerated in CK and PK (Fig. 4C). AP exhibited marked interannual variation, with continuous accumulation under PK reaching more than 1.2-fold higher than CK, whereas CK and NP declined by 60–70% by 2021 relative to 2014 (Fig. 4D). AK also differed significantly among treatments (*p* < 0.001), with substantial accumulation under PK and NK fertilization (Fig. 4E).

**Fig. 5:**
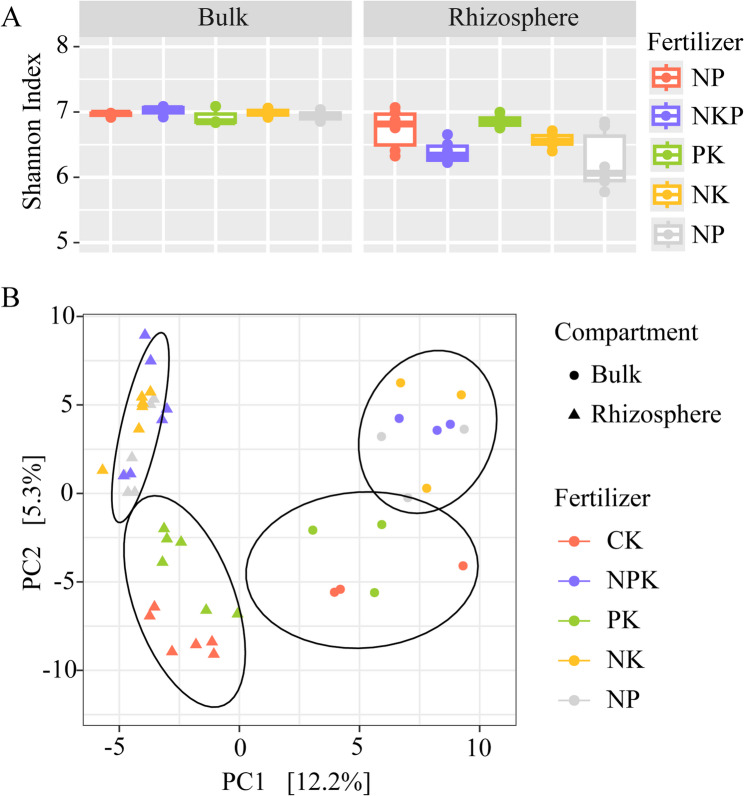
Bacterial alpha diversity (Shannon index) (**A**) and principal component analysis of bacterial communities (**B**) in bulk and rhizosphere soils under different fertilizer treatments: CK, NPK, PK, NK, and NP

Under varying fertilizer treatments, no significant differences in bacterial alpha diversity (Shannon index) were observed in the bulk soil samples (Fig. 5A). However, rhizosphere samples displayed significant variation (*p* < 0.05). More specifically, the highest bacterial diversity was detected in rhizosphere samples treated with PK fertilizers, whereas the lowest diversity occurred with NP fertilization (Fig. 5A). Notably, rhizosphere samples without N supplementation (CK and PK treatments) exhibited greater bacterial diversity compared to those receiving N inputs.

Both the sample compartment (bulk soil vs. rhizosphere soil) and fertilizer treatment significantly influenced bacterial community structure (*p* = 0.001) (Table S2). The primary driver of community separation was the sample compartment, with distinct clustering observed between bacterial communities in bulk soil and rhizosphere soil (Fig. 5B). Within each sample compartment, fertilizer treatments further shaped the bacterial community structure, with samples receiving N fertilizer showing pronounced separation from those without N supplementation (CK and PK) (Fig. 5B).

### Specific maize rhizosphere bacteria taxa enriched under different fertilization treatments

**Fig. 6:**
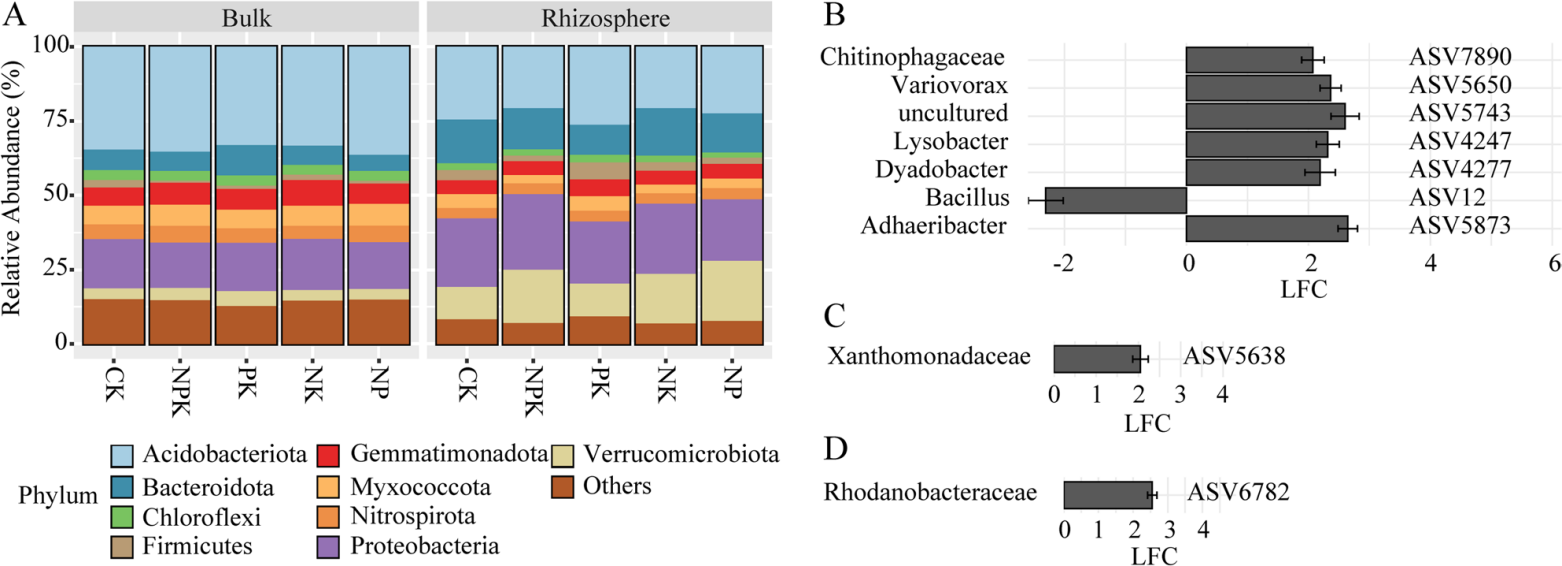
Relative abundance of bacterial phyla in bulk and rhizosphere soils under different fertilizer treatments: CK, NPK, PK, NK, NP (**A**) and log fold change (LFC) abundance of rhizosphere bacterial taxa and ASVs showing differential responses to N (**B**), P (**C**), and K (**D**) fertilization. Positive values indicate higher bacterial abundance in soils supplied with N, P, K, while negative values indicate higher bacterial abundance in soils without the respective fertilizer

Similar bacterial phyla were identified in both the bulk and rhizosphere soils of maize, primarily comprising Acidobacteriota (23–35%), Bacteroidota (10%-30%), Gemmatimonadota (12–25%), Myxococcota (10–25%), Nitrospirota (8–12%), Proteobacteria (40–60%), and Verrucomicrobiota (8–50%) (Fig. 6A). Acidobacteriota, Gemmatimonadota, and Myxococcota showed higher relative abundance in bulk soil compared to rhizosphere soil, whereas Proteobacteria and Verrucomicrobiota were enriched in the rhizosphere. Fertilization effects on bacterial abundance were more pronounced in the rhizosphere than in bulk soil (Fig. 5B). In the rhizosphere soil, N-fertilized treatments (NPK, NK, and NP) exhibited higher relative abundance of Verrucomicrobiota but lower abundance of Acidobacteriota and Myxococcota compared with soils without N supplementation (CK and PK).

Analyses of the influence of specific fertilizer types (N, P, and K) on rhizosphere bacterial communities revealed significant variations in the abundance of specific bacteria amplicon sequence variants (ASVs). A total of 68 ASVs exhibited significantly different relative abundances under N-supplied (+ N) compared to N-deficient (-N) soils. Similarly, 24 ASVs showed significant differences under varying P-supplied (+ P vs. -P), and another 24 ASVs were identified under different K fertilizer conditions (+ K vs. -K). For bacterial taxa with a Log-Fold Change (LFC) > 2, *Chitinophagaceae*,* Variovorax*,* Microscillaceae*,* Lysobacter*,* Dyadobacter*, and *Adhaeribacter* were more abundant in N-supplied soils, while *Bacillus* were enriched in soils without N addition (Fig. 4). Besides, *Xanthomonadaceae* exhibited higher abundance in P-supplied soils, while *Rhodanobacteraceae* was more prevalent in K-supplied soils (Fig. 6).

Based on plant and soil measurements obtained in 2021, Spearman correlation analysis revealed that the rhizosphere bacteria ASV12 (*Bacillus*), enriched under N-depleted conditions, was significantly negatively correlated with several plant and soil properties. Specifically, *Bacillus* abundance was inversely correlated with grain yield, grain nitrogen content, shoot nitrogen content, shoot and root dry weights, root total volume, root average diameter, root surface area, and root total length (*p* < 0.001). On the contrary, bacteria *Lysobacter* (ASV4247), *Dyadobacter* (ASV4277), *Variovorax* (ASV5650), *Microscillaceae* (ASV5743) enriched in N-adequate conditions, exhibited positive correlations with these same plant and soil properties (*p* < 0.05) (Fig. S4). In addition, rhizosphere bacteria enriched in P abundant conditions (ASV5638 *Xanthomonadaceae*) showed positive correlations with grain yield and most plant shoot and root growth properties, while bacteria (ASV 6782 *Rhodanobacteraceae*) varied under different K supplies did not show significant correlations with any measured plant or soil traits (Fig. S3).

## Discussion

### Responses of maize growth and soil nutrients to long-term NPK fertilization

This study demonstrates that balanced fertilization with nitrogen (N), phosphorus (P), and potassium (K) markedly enhances maize growth, grain yield, and the recruitment of the rhizosphere microbiome. Among these macronutrients, N exerted the strongest influence on plant performance. Severe reductions in shoot and root biomass, as well as grain yield, were observed under N-limited conditions (CK and PK) compared with treatments receiving N fertilization (NPK, NK, and NP) (Figs. 1, 2 and 3). These results underscore the critical role of adequate N supply in sustaining maize growth and productivity. Our findings align with previous research indicating that N and P deficiencies impose more stringent limitations on maize yields and nutrient-use efficiency than K deficiency [38], and that N fertilization significantly promotes dry matter accumulation, grain yield, and root system architecture [14, 39]. Despite the dominant role of nitrogen, balanced NPK fertilization produced the highest grain N and P concentrations, shoot and root biomass, and grain yield compared with the unfertilized control (CK). This emphasizes the necessity of nutrient balance and supports previous reports on the synergistic effects of N, P, and K in sustaining crop productivity [38, 40].

Long-term field experiments provide essential insights into soil nutrient dynamics that short-term studies cannot capture. Such longitudinal data are particularly valuable for assessing how sustained nutrient voids alter soil pools and inherent fertility over time [41–43]. In this study, decade-long deficiencies of N, P, or K led to distinct nutrient depletion in the purple soil (Regosols) (Fig. 4). Nitrogen deficiency sharply reduced soil available N, highlighting its role not only in immediate plant uptake but also in maintaining soil nitrogen reservoirs and promoting organic matter synthesis. Furthermore, the inherently low P background of purple soils led to the rapid exhaustion of soil available P under P-deficient regime, while long-term K deficiency exhausted soil available K, despite K-rich parent materials. These outcomes are consistent with earlier findings that sustainable maize yields require balanced nutrient supply, since NPK fertilization improves soil aggregate stability, reduces N and P losses, and promotes yield stability by enhancing nutrient cycling [28, 38].

Purple soils in Southwest China are generally characterized by low organic matter and nitrogen but are relatively rich in potassium. This stands in contrast to other major agricultural soils, such as Phaeozem (black soils) and Cambisol, which are typically more fertile and show more robust increases in organic matter under conventional fertilization [23]. The limited accumulation of organic matter and N in purple soils can be attributed to subtropical climate conditions, which accelerate organic matter decomposition and nutrient loss; intensive double-cropping systems, which deplete soil fertility; and the prevalence of sloped farmland with weak structure, which increases susceptibility to erosion. Despite these constraints, long-term NPK fertilization significantly increased available nitrogen (AN) and phosphorus (AP) compared with the unfertilized control (Fig. 4). However, AN still exhibited a gradual decline over time, indicating that conventional fertilization alone may not fully prevent nutrient depletion or soil degradation in purple soils. This pattern likely reflects the combined effects of high crop nutrient demand, rapid organic matter turnover, and environmental conditions that limit nutrient retention. These findings suggest that while NPK fertilization can sustain crop productivity, additional management strategies (e.g., organic amendments or soil conservation practices) may be required to improve long-term soil fertility and sustainability in purple soils.

Building on a decade of long-term fertilization data, soil nutrient monitoring, and maize growth measurements, we analyzed the root microbiome to assess the cumulative effects of prolonged fertilizer treatments. These results demonstrate how long-term nutrient management shapes rhizosphere microbial communities and highlight the importance of sustained fertilization strategies for influencing soil–plant–microbiome interactions in purple soils.

### Responses of rhizosphere microbial diversity to NPK fertilization

The rhizosphere microbiome is a key driver of nutrient cycling, plant health, and soil fertility through processes such as N fixation, P solubilization, and organic matter decomposition [44, 45]. Consistent with prior research, bacterial alpha diversity was lower in the rhizosphere than in the bulk soil (Fig. 5A), highlighting the strong influence of plant-mediated recruitment of root-associated microbiota [46, 47].

Fertilization regimes further altered rhizosphere diversity and community structure. Higher bacterial Shannon diversity was observed in rhizosphere without N fertilization (CK and PK), while N-fertilized soils (NPK, NP, and NK) exhibited lower diversity (Fig. 5A). Community composition also clustered distinctly between N-supplied and N-deficient soils (Fig. 5B). These results align with previous studies reporting reduced rhizosphere diversity under N fertilization [48, 49], as well as shifts in microbial structure in bulk soils following long-term N input [50]. Comparative analyses across different soil types, such as Phaeozem, Cambisol, and Acrisol, have shown that while mineral fertilizers can shift community structures, the combined application of chemical fertilizers and organic amendments is often required to significantly increases bacterial abundance and diversity [23].

However, contrasting evidence also exists. Some studies report increased microbial diversity with N fertilization, particularly under long-term or slow-release N fertilizer applications [16, 51]. Other findings suggest limited rhizosphere responses, with N effects confined to root-associated microbiota [52]. Such discrepancies likely reflect variations in: (i) host traits such as plant genotype, root architecture, and exudation [53, 54]; (ii) soil properties including pH, nutrient availability, and texture [55, 56]; and (iii) agricultural practices such as fertilizer form, rate, and placement [57–59]. These findings underscore the complexity of between fertilization-microbiome interactions and highlight the need for tailored fertilization strategies that account for plant-soil-microbe feedbacks.

### Functional implications of rhizosphere taxa under NPK fertilization

We profiled the bacterial community composition in the maize rhizosphere and identified dominant taxa at the phylum level, including Acidobacteriota, Bacteroidota, Chloroflexi, Firmicutes, Gemmatimonadota, Myxococcota, Nitrospirota, Proteobacteria, and Verrucomicrobiota (Fig. 6A). These findings are consistent with previous studies reporting that maize rhizosphere communities are typically dominated by a similar set of bacterial phyla across different soils and regions. This pattern suggests a broadly conserved rhizosphere microbial structure associated with maize cultivation under comparable environmental conditions and management practices [60].

At the genus and family levels, rhizosphere bacterial taxa such as *Chitinophagaceae*,* Variovorax*,* Microscillaceae*,* Lysobacter*,* Dyadobacter*, and *Adhaeribacter* were more abundant in nitrogen (N)-amended soils (Fig. 6), with positive correlations detected between maize grain yield and the abundance of *Variovorax* (ASV5650), *Microscillaceae* (ASV5743), *Lysobacter* (ASV4247), and *Dyadobacter* (ASV4277) (Fig. S4). Among these taxa: *Chitinophagaceae* exhibited higher abundance in maize rhizospheres under normal and reduced NPK fertilization (NP, NK, and PK) compared to the unfertilized control. This enrichment likely contributes to rhizosphere health; for instance, *Chitinophagaceae* has been linked to enhanced photosynthesis and yield formation in sorghum [61]. Additionally, members of this family in bulk soil harbor genes involved in P mineralization, suggesting a potential synergistic interplay between N and P in regulating microbial community functions [62].

*Variovorax*, specifically strain P1R9, has been reported to improve wheat germination and plant growth under salt stress [63]. *Variovorax* strains isolated from maize rhizospheres exhibit 1-aminocyclopropane-1-carboxylate (ACC) deaminase activity, which can break down ACC into ammonia and promote plant growth [64, 65]. *Microscillaceae*, identified as potential nitrifying bacteria [66], have been implicated in shaping the rhizosphere microbiome in long-term mono cropping systems [67]. A negative correlation between *Microscillaceae* abundance and phosphatase activity in maize rhizosphere soils further underscores their role in nutrient cycling [38]. *Lysobacter* species, abundant in carbonate-rich, alkaline soils (pH 8.5), enhance nitrogen transport efficiency, nitrogen fixation, and organic matter decomposition while promoting plant growth [16, 46, 68, 69]. *Dyadobacter*, previously isolated from sterilized maize stems [70], is believed to fix atmospheric nitrogen, thus supporting plant growth [71]. *Adhaeribacter* abundance increases with organic matter addition in rhizospheres, as observed in *Populus* systems. Some species secrete enzymes that decompose organic substances, facilitating nutrient turnover [72, 73]. Collectively, these rhizosphere taxa, particularly *Variovorax*, *Microscillaceae*,* Lysobacter*, and *Dyadobacter*, appear to play significant roles in promoting maize growth and productivity. Future studies should focus on elucidating the mechanistic basis of these interactions to optimize their potential for agricultural applications.

On the contrary, *Bacillus* species in the rhizosphere were enriched in N-deficient soils (Fig. 6A). Several *Bacillus* species have been identified as N-fixing bacteria in both the phyllosphere [74] and rhizosphere [75] of maize. Under N-limited conditions, inoculation with *Bacillus* strains has been shown to enhance photosynthetic rate and efficiency, thereby promoting maize growth [76, 77]. These findings underscore the potential role of rhizosphere *Bacillus* species in N cycling and plant growth promotion, particularly under nutrient-limited conditions. However, the observed enrichment of *Bacillus* (ASV12) under N-depleted conditions could also have adverse effects on maize growth and yield. This aligns with findings in rice, where specific *Bacillus* strains negatively impacted plant performance [78]. These contrasting effects indicate that the functional role of *Bacillus* species in the rhizosphere may depend on their specific strains and interactions with the host plant, highlighting the complexity of microbial contributions to plant-microbe interactions under varying nutrient conditions [44].

In addition, *Xanthomonadaceae* were enriched in P-supplied soils, whereas *Rhodanobacteraceae* were more prevalent in K-supplied soils (Fig. 6A). *Xanthomonadaceae* have been recognized as a dominant microbial group in the rhizoplane [79] and rhizosphere of maize [47]. Their relative abundance has been reported to increase in response to root exudates [80], manure amendments [81], and nitrogen addition [82], suggesting a strong preference for nutrient-rich environments. *Rhodanobacteraceae* in the maize rhizosphere have been shown to play key roles in carbon and N cycling. Specifically, they enhance processes such as chemoheterotrophy and N fixation while simultaneously inhibiting fermentation and nitrate reduction, particularly under conditions such as straw mulch addition [40]. These findings highlight the functional specialization of *Rhodanobacteraceae* and their potential contributions to nutrient transformations in agroecosystems.

While the present study identified fertilizer-responsive bacterial taxa and their associations with plant performance, future research utilizing targeted qPCR and shotgun metagenomics is needed to provide higher functional resolution. Such approaches will help clarify the mechanistic contributions of these microbial groups to maize nutrient uptake and soil fertility under long-term management in purple soil systems.

## Conclusion

This 10-year field experiment demonstrates that balanced fertilization with nitrogen (N), phosphorus (P), and potassium (K) is essential for sustaining maize productivity in purple soils, a fragile and inherently low-fertility soil type. Among the three macronutrients, nitrogen emerged as the most critical driver of maize growth, biomass accumulation, and grain yield, yet balanced NPK fertilization consistently outperformed partial or single nutrient applications. Over the decade-long study, sustained nutrient deficiencies led to marked declines in soil available N, P, and K, whereas NPK fertilization improved soil nutrient availability and promoted yield stability. However, soil available nitrogen (AN) was not fully maintained over time, indicating that conventional fertilization alone may be insufficient to prevent long-term nutrient depletion in this system.

Beyond abiotic factors, long-term fertilization significantly restructured the maize rhizosphere microbiome. Nitrogen supply was the primary determinant of bacterial alpha diversity and community composition. Specific taxa, including *Variovorax*, *Microscillaceae*, *Lysobacter*, and *Dyadobacter*, were positively associated with maize performance under N-supplied conditions, suggesting their role as key beneficial players in the rhizosphere. Conversely, *Bacillus* was enriched under N-deficient conditions, though its negative correlation with yield indicates a context-dependent functional role.

Overall, this study underscores the dual importance of balanced fertilization in optimizing crop performance while simultaneously fostering beneficial plant–microbe–nutrient interactions. These findings provide a robust scientific foundation for developing targeted nutrient management strategies tailored to the purple soils of Southwest China, demonstrating how long-term management practices can secure agricultural sustainability by harmonizing soil health and microbial function.

## Supplementary Information

Supplementary Material 1.

## Data Availability

The datasets generated during the current study are available in the NCBI repository(PRJNA1417479).
