# Supplementary material for: Balanced NPK fertilization enhances maize yield and shapes rhizosphere bacterial communities in purple soil: evidence from a ten-year field experiment
Source: BMC Microbiol. 2026 May 12;26:594. doi: 10.1186/s12866-026-05142-0 (PMC13335213; doi:10.1186/s12866-026-05142-0)
Supplement: Supplementary file 1 — Supplementary Material 1. [file 12866_2026_5142_MOESM1_ESM.docx]

# **Supplementary**

**Table S1**. Soil properties (pH, organic matter (OM), available N (AN), available P (AP), and available K (AK)) in bulk and rhizosphere soils under different fertilizer treatments (CK, NPK, PK, NK, NP). Values are presented as mean ± SE, with different letters indicating significant differences at p < 0.05.

| Compartment | Treatment | pH | OM (mg/kg) | AN (mg/kg) | AP (mg/kg) | AK (mg/kg) |
| --- | --- | --- | --- | --- | --- | --- |
| Bulk | CK | 8.58±0.04ab | 9.94±1.27a | 52±5.5b | 3.70±0.20c | 111±7.0b |
|  | NPK | 8.61±0.05a | 11.27±0.91a | 64±9.2a | 6.47±1.11ab | 143.7±12.1a |
|  | PK | 8.52±0.02ab | 10.44±0.53a | 53±1.7b | 7.93±1.39a | 142±19.7a |
|  | NK | 8.48±0.04b | 10.77±0.46a | 55±3.5ab | 3.87±0.45c | 136±6.2a |
|  | NP | 8.53±0.11ab | 10.23±0.21a | 58±1.5ab | 5.27±0.57bc | 105±3.2b |
| Rhizosphere | CK | 8.64±0.02a | 10.36±0.21ab | 59±0.9a | 3.80±0.10bc | 104±1c |
|  | NPK | 8.60±0.02a | 11.43±0.23a | 63±0.8a | 5.93±0.43ab | 133±3b |
|  | PK | 8.59±0.01ab | 11.00±0.31ab | 59±2.6a | 8.10±1.19a | 157±9a |
|  | NK | 8.54±0.01b | 10.96±0.44ab | 61±3.3a | 3.10±0.13c | 143±6ab |
|  | NP | 8.61±0.01a | 9.88±0.32b | 55±1.8a | 5.70±0.23b | 93±2c |

**Table S2.** Permutational multivariate analysis of variance (PERMANOVA) of bacterial beta diversity explained by fertilizer treatment and soil compartment (bulk soil and rhizopshere soil).

|  | R^2^ | F | *P* value |
| --- | --- | --- | --- |
| Fertilizer | 0.122 | 1.589 | 0.001 |
| Compartment | 0.113 | 5.899 | 0.001 |
| Fertilizer:Compartment | 0.091 | 1.181 | 0.031 |


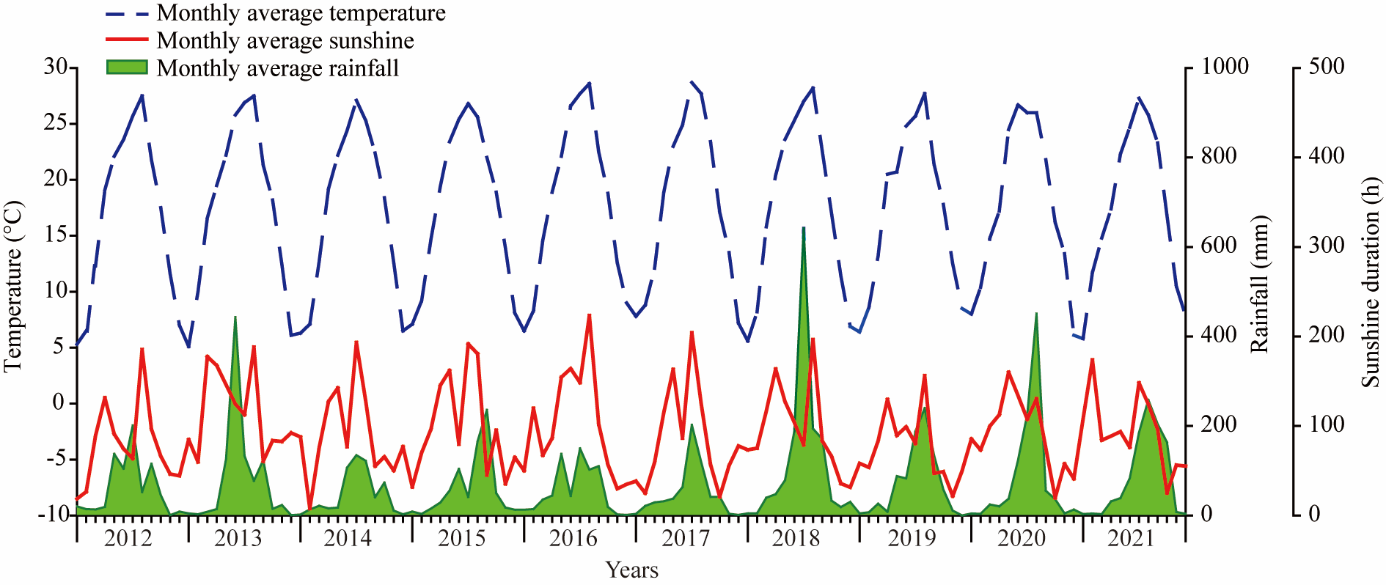


**Fig. S1** Monthly average temperature, sunshine, and rainfall at the experimental site from 2012 to 2021.

**Fig. S2** Maize yield from 2012 to 2021 under different fertilizer treatments: no fertilizer (CK), nitrogen and phosphorus fertilizers (NP), nitrogen and potassium fertilizers (NK), phosphorus and potassium fertilizers (PK), and nitrogen, phosphorus, and potassium fertilizers (NPK). Values followed by different lowercase letters indicate significant difference among treatments at *P* < 0.05 (F-test). Bars represent means ± SD of three replicates (n = 3).


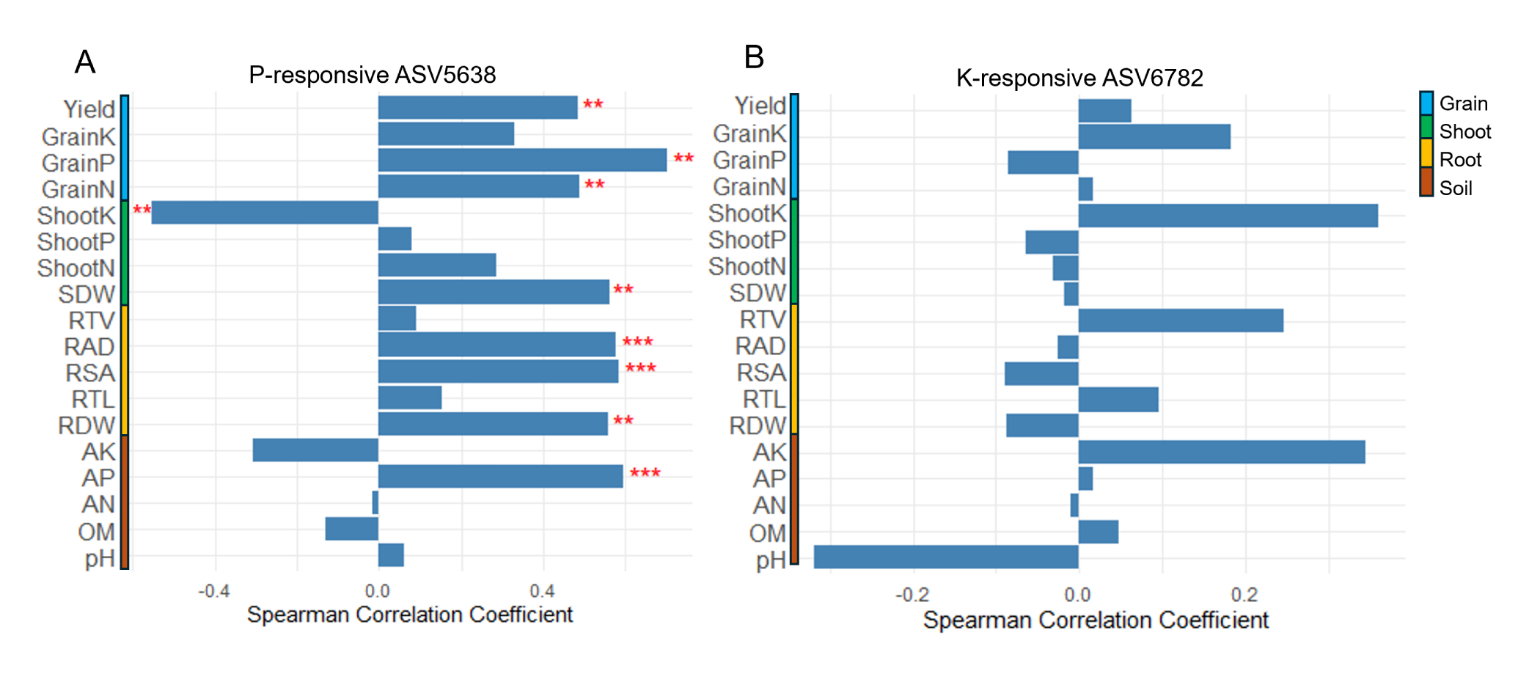


**Fig. S3** Spearman correlations between key plant and soil properties and nutrient-responsive rhizosphere bacterial ASVs: ASV5638 under varying P supply (A) and ASV6782 under varying K supply (B). Correlations are shown for grain traits (yield, N, P, K content), shoot traits (shoot dry weight (SDW), shoot N content, shoot P content, shoot K content), root traits (root dry weight (RDW), root total length (RTL), root surface area (RSA), root average diameter (RAD), root total volume (RTV)), and soil properties (pH, organic matter (OM), available N (AN), available P (AP), and available K (AK)). Positive correlations are represented in orange, and negative correlations in purple, with Spearman correlation coefficients displayed. Statistical significance is indicated by asterisks: *** *p* < 0.001, ** *p* < 0.01, and * *p* < 0.05 (n = 3).


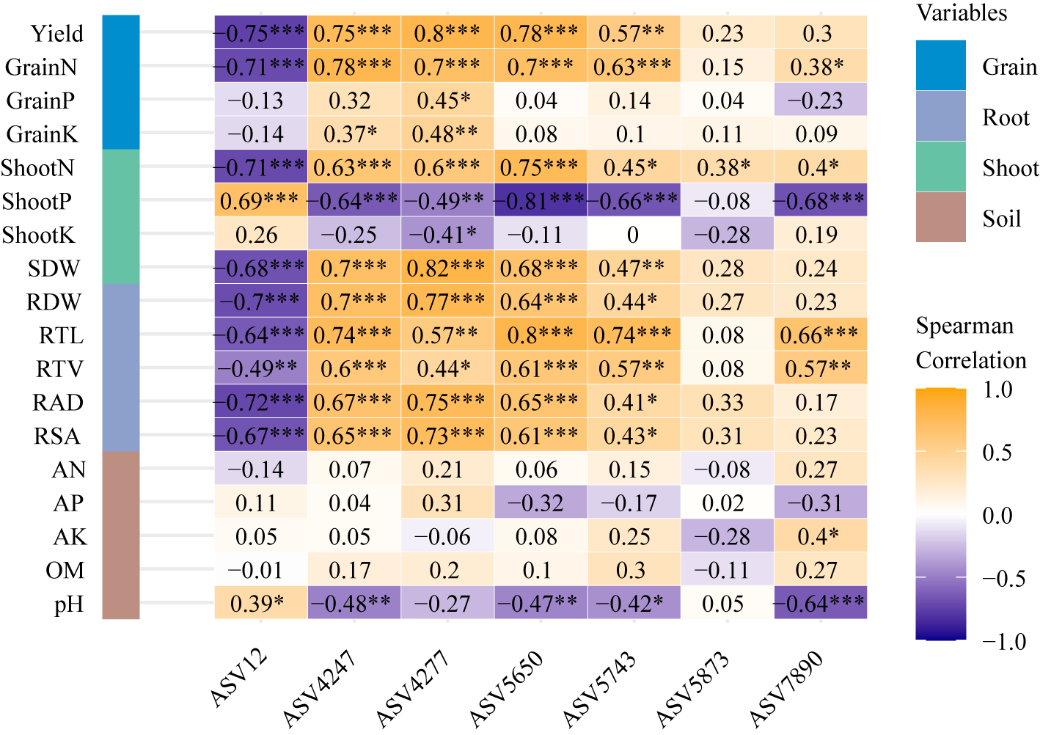


**Fig. S4** Spearman correlations between differentially abundant rhizosphere bacterial ASVs under varying nitrogen (N) supplies and key plant and soil properties. Correlations are shown for grain traits (yield, N, P, K content), shoot traits (shoot dry weight (SDW), shoot N content, shoot P content, shoot K content), root traits (root dry weight (RDW), root total length (RTL), root surface area (RSA), root average diameter (RAD), root total volume (RTV)), and soil properties (pH, organic matter (OM), available N (AN), available P (AP), and available K (AK)). Positive correlations are represented in orange, and negative correlations in purple, with Spearman correlation coefficients displayed. Statistical significance is indicated by asterisks: * *p* < 0.05, ** *p* < 0.01, and *** *p* < 0.001 (n = 3).
